# Supplementary material for: Local mitochondrial physiology defined by mtDNA quality guides purifying selection
Source: PLoS Genet. 2026 Jan 9;22(1):e1011836. doi: 10.1371/journal.pgen.1011836 (PMC12810922; doi:10.1371/journal.pgen.1011836)
Supplement: S1 Table — (DOCX) [file pgen.1011836.s001.docx]

Table 1 - Yeast Strains used in this study

| Name | Short description | Mating type | Genotype | mtDNA | Source |
| --- | --- | --- | --- | --- | --- |
| yCJ084 | ATP6-NG | Mat alpha | leu2-3,112 can1-100 ura3-1  his3-11,15 | ATP-NG | Jakubke et al. (2021) |
| yRR120 | *∆arg8*::HIS3  *∆cob*::ARG8M | Mat a | ade2-1 his3-11,15 trp1-1 leu2-3,112  ura3-1 CAN1 arg8::HIS3 | *∆cob*::ARG8M  Intronless mtDNA | Jakubke et al. (2021) |
| yCO355 | *∆arg8*::HIS3  *∆cox2*::ARG8M | Mat a | ade2-1 his3-11 15 trp1-1 leu2-3 112 ura3-1 CAN1 arg8::HIS3 | *∆cox2*::ARG8M | Gruschke et al. (2011) |
| yCO391 | *∆arg8* | Mat a | ade2-1 his3-11,15 trp1-1 leu2-3,112  ura3-1 CAN1 arg8::HIS3 | Intronless mtDNA | Gruschke et al. (2011) |
| yCO534 | WT | Mat a | ade2-1 his3-11,15 trp1-1 leu2-3,112 ura3-1 CAN1 arg8::HIS3 |  | Rak et al. (2007) |
| yCO535 | *∆arg8*::HIS3  *∆atp6*::ARG8M | Mat a | ade2-1 his3-11,15 trp1-1 leu2-3,112 ura3-1 CAN1 arg8::HIS3 | *∆atp6*::ARG8M | Rak et al. (2007) |
| yFT310 | ATP6-NG pdr5::Hygromycin | Mat alpha | leu2-3,112 can1-100 ura3-1 his3-11,15 ATP6-NeonGreen ∆pdr5::Hygromycin | ATP-NG | This study |
| yCO354 | *∆arg8*::HIS3  *∆cob*::ARG8M | Mat alpha | ade2-1 his3-11,15 trp1-1 leu2-3,112  ura3-1 CAN1 arg8::HIS3 | *∆cob*::ARG8M  intronless mtDNA | Gruschke et al. (2011) |
| ySS935 | *∆arg8*::HIS3  *∆atp6*::ARG8M | Mat alpha | ade2-1 his3-11,15 trp1-1 leu2-3,112 ura3-1 CAN1 arg8::HIS3 | *∆atp6*::ARG8M | Schrott et al. (2023) |
| ySS936 | *∆cox2*::ARG8M | Mat alpha | ade2-1 his3-11 15 trp1-1 leu2-3 112 ura3-1 CAN1 arg8::HIS3 | *∆cox2*::ARG8M | Schrott et al. (2023) |
| yFT358 | ATP6-NG ∆atp20 | Mat alpha | leu2-3,112 can1-100 ura3-1 his3-11,15 ∆atp20::hphNT1 | ATP6-NeonGreen | This study |
| yFT359 | *∆arg8*::HIS3  *∆cob*::ARG8M  ∆atp20 | Mat a | ade2-1 his3-11,15 trp1-1 leu2-3,112 ura3-1 CAN1 arg8::HIS3 ∆atp20::hphNT1 | *∆cob*::ARG8M | This study |
| yFT360 | arg8::HIS3 cox2::ARG8M  ∆atp20 | Mat a | ade2-1 his3-11 15 trp1-1 leu2-3 112 ura3-1 CAN1 arg8::HIS3 ∆atp20::hphNT1 | *∆cox2*::ARG8M | This study |
| yFT361 | ∆atp20 | Mat a | ade2-1 his3-11,15 trp1-1 leu2-3,112 ura3-1 CAN1 arg8::HIS3 ∆atp20::hphNT1 |  | This study |
| yFT362 | *∆arg8*::HIS3  *∆atp6*::ARG8M  ∆atp20 | Mat a | ade2-1 his3-11,15 trp1-1 leu2-3,112 ura3-1 CAN1 arg8::HIS3 ∆atp20::HphNT1 | *∆atp6*::ARG8M | This study |
| yCO841 | *∆arg*::HIS3  *∆atp20* | Mat a | ade2-1 his3-11,15 trp1-1 leu2-3,112 ura3-1 CAN1 arg8::HIS3 ∆atp20::hphNT1 | intronless mtDNA |  |
| yFT215 | Pgk1-Su9-QUEEN-2M | Mat alpha | leu2-3,112 trp1-1 can1-100 ura3-1 ade2-1 his3-11,15 LEU- Pgk1-Su9-QUEEN-2M |  | This study |
| yFT216 | *∆arg8*::HIS3  *∆cob*::ARG8M  Pgk1-Su9-QUEEN-2M | Mat alpha | ade2-1 his3-11,15 trp1-1 leu2-3,112 ura3-1 CAN1 arg8::HIS3 LEU-Pgk1-Su9-QUEEN-2M | *∆cob*::ARG8M intronless mtDNA | This study |
| yFT217 | *∆arg8*::HIS3  *∆cox2*::ARG8M  Pgk1-Su9-QUEEN-2M | Mat a | ade2-1 his3-11 15 trp1-1 leu2-3 112 ura3-1 CAN1 arg8::HIS3 LEU-Pgk1-Su9-QUEEN-2M | *∆cox2*::ARG8M | This study |
| yFT218 | *∆arg8*  Pgk1-Su9-QUEEN-2M | Mat a | ade2-1 his3-11,15 trp1-1 leu2-3,112 ura3-1 CAN1 arg8::HIS3 LEU-Pgk1-Su9-QUEEN-2M::URA3 | intronless mtDNA | This study |
| yFT219 | *∆arg8*::HIS3  *∆atp6*::ARG8M Pgk1-Su9-QUEEN-2m | Mat a | ade2-1 his3-11,15 trp1-1 leu2-3,112 ura3-1 CAN1 arg8::HIS3 LEU-Pgk1-Su9-QUEEN-2M | *∆atp6*::ARG8M | This study |
| yFT230 | Pgk1-Su9-QUEEN-2m | Mat alpha | ade2-1 his3-11,15 trp1-1 leu2-3,112 ura3-1 CAN1 arg8::HIS3 LEU-Pgk1-Su9-QUEEN-2M |  | This study |
| yFT232 | Pgk1-Su9-QUEEN-2m | Mat alpha | ade2-1 his3-11,15 trp1-1 leu2-3,112 ura3-1 CAN1 arg8::HIS3 LEU-Pgk1-Su9-QUEEN-2M |  | This study |
| yFT233 | Pgk1-Su9-QUEEN-2m | Mat a | Mata ade2-1 his3-11,15 trp1-1 leu2-3,112 ura3-1 CAN1 arg8::HIS3 LEU-Pgk1-Su9-QUEEN-2M |  | This study |
| yFT221 | *∆arg8*::HIS3  *∆cob*::ARG8M  Su9-mNeonGreen | Mat alpha | ade2-1 his3-11,15 trp1-1 leu2-3,112 ura3-1 CAN1 arg8::HIS3  HO-Su9-mNeonGreen::KanMX6 | *∆cob*::ARG8M  intronless mtDNA | This study |
| yFT222 | *∆arg8*::HIS3  *∆cox2*::ARG8M  Su9-mNeonGreen | Mat a | ade2-1 his3-11 15 trp1-1 leu2-3 112 ura3-1 CAN1 arg8::HIS3  HO-Su9-mNeonGreen::kanMX6 | *∆cox2*::ARG8M | This study |
| yFT223 | *∆arg8*  Su9-mNeonGreen | Mat a | ade2-1 his3-11,15 trp1-1 leu2-3,112 ura3-1 CAN1 arg8::HIS3  HO-Su9-mNeonGreen ::kanMX6 | Intronless mtDNA | This study |
| yFT224 | *∆arg8*::HIS3  *∆atp6*::ARG8M  Su9-mNeonGreen | Mat a | ade2-1 his3-11,15 trp1-1 leu2-3,112 ura3-1 CAN1 arg8::HIS3 HO-Su9-mNeonGreen ::kanMX6 | ∆atp6::ARG8 | This study |
| yFT234 | Su9-mNeonGreen | Mat alpha | Mata ade2-1 his3-11,15 trp1-1 leu2-3,112 ura3-1 CAN1 arg8::HIS3 HO-Su9-mNeonGreen ::kanMX6 |  | This study |
| yFT235 | Su9-mNeonGreen | Mat a | leu2-3,112 trp1-1 can1-100 ura3-1 ade2-1 his3-11,15 HO-Su9-mNeonGreen ::kanMX6 |  | This study |
| yFT237 | Su9-mNeonGreen | Mat a | ade2-1 his3-11,15 trp1-1 leu2-3,112 ura3-1 CAN1 arg8::HIS3  HO-Su9-mNeonGreen ::kanMX6 |  | This study |
| yFT005 | Pcup1-Su9-3xNG-LacI--PGK1-Su9-mKate | Mat alpha | leu2-3,112 can1-100 ura3-1 his3-11,15 Pcup1-Su9-3xNG-LacI--PGK1-Su9-mKate | LacO-mtDNA | This study |
| yFT296 | ATP6-NeonGreen ∆cox4 | Mat alpha | leu2-3,112 can1-100 ura3-1 his3-11,15 ATP6-NeonGreen ∆cox4::HphNT1 |  | This study |
| yFT298 | arg8::HIS3  cox2::ARG8M ∆cox4 | Mat a | ade2-1 his3-11 15 trp1-1 leu2-3 112 ura3-1 CAN1 arg8::HIS3 ∆cox4::HphNT1 | *∆*cox2::ARG8M | This study |
| yFT299 | ∆cox4 | Mat a | ade2-1 his3-11,15 trp1-1 leu2-3,112 ura3-1 CAN1 arg8::HIS3 ∆cox4::HphNT1 |  | This study |
| yFT300 | arg8::HIS3  atp6::ARG8M ∆cox4 | Mat a | ade2-1 his3-11,15 trp1-1 leu2-3,112 ura3-1 CAN1 arg8::HIS3 ∆cox4::HphNT1 | *∆atp6*::ARG8M | This study |
| yFT367 | arg8::HIS3  cob::ARG8M ∆cox4 | Mat a | ade2-1 his3-11,15 trp1-1 leu2-3,112 ura3-1 CAN1 arg8::HIS3 ∆cox4::HphNT1 | *∆cob*::ARG8M  Intronless mtDNA | This study |
| yFT326 | ATP6-NeonGreen ∆rip1 | Mat alpha | leu2-3,112 can1-100 ura3-1 his3-11,15 ∆rip1::HphNT1 | ATP6-NeonGreen | This study |
| yFT329 | arg8::HIS3  cox2::ARG8M ∆rip1 | Mat a | ade2-1 his3-11 15 trp1-1 leu2-3 112 ura3-1 CAN1 arg8::HIS3 ∆rip1::HphNT1 | *∆cox2*::ARG8M | This study |
| yFT331 | ∆rip1 | Mat a | ade2-1 his3-11,15 trp1-1 leu2-3,112 ura3-1 CAN1 arg8::HIS3 ∆rip1::HphNT1 |  | This study |
| yFT332 | arg8::HIS3  atp6::ARG8M ∆rip1 | Mat a | ade2-1 his3-11,15 trp1-1 leu2-3,112 ura3-1 CAN1 arg8::HIS3 ∆rip1::HphNT1 | *∆atp6*::ARG8M | This study |
| yFT357 | arg8::HIS3  cob::ARG8M ∆rip1 | Mat a | ade2-1 his3-11,15 trp1-1 leu2-3,112 ura3-1 CAN1 arg8::HIS3 ∆rip1::HphNT1 | *∆cob*::ARG8M  Intronless mtDNA | This study |
| yFT368 | Su9-mNeonGreen ∆cox4 | Mat alpha | leu2-3,112 trp1-1 can1-100 ura3-1 ade2-1 his3-11,15 HO-Su9-mNeonGreen-KanMX6 ∆cox4::HphNT1 |  | This study |
| yFT369 | Su9-mNeonGreen ∆cob::ARG8 ∆cox4 | Mat alpha | ade2-1 his3-11,15 trp1-1 leu2-3,112 ura3-1 CAN1 arg8::HIS3 HO-Su9-mNeonGreen-KanMX6 ∆cox4::HphNT1 | cob::ARG8M  intronless mtDNA | This study |
| yFT370 | Su9-mNeonGreen ∆cox2::ARG8 ∆cox4 | Mat a | ade2-1 his3-11 15 trp1-1 leu2-3 112 ura3-1 CAN1 arg8::HIS3 HO-Su9-mNeonGreen-KanMX6 ∆cox4::HphNT1 | ∆*cox2*::ARG8 | This study |
| yFT371 | Su9-mNeonGreen ∆cox4 | Mat a | ade2-1 his3-11,15 trp1-1 leu2-3,112 ura3-1 CAN1 arg8::HIS3 HO-Su9-mNeonGreen-KanMX6::URA3 ∆cox4::HphNT1 | Intronless mtDNA | This study |
| yFT372 | Su9-mNeonGreen ∆atp6::ARG8 ∆cox4 | Mat a | ade2-1 his3-11,15 trp1-1 leu2-3,112 ura3-1 CAN1 arg8::HIS3 ∆atp6::ARG8 HO-Su9-mNeonGreen-KanMX6 ∆cox4::HphNT1 |  | This study |
| yFT373 | Su9-mNeonGreen ∆cox4 | Mat alpha | Mata ade2-1 his3-11,15 trp1-1 leu2- Mat a 3,112 ura3-1 CAN1 arg8::HIS3 HO-Su9-mNeonGreen-KanMX6 ∆cox4::HphNT1 |  | This study |
| yFT374 | Su9-mNeonGreen ∆cox4 | Mat a | leu2-3,112 trp1-1 can1-100 ura3-1 ade2-1 his3-11,15 HO-Su9-mNeonGreen-KanMX6 ∆cox4::HphNT1 |  | This study |
| yFT375 | Su9-mNeonGreen ∆cox4 | Mat alpha | ade2-1 his3-11,15 trp1-1 leu2-3,112 ura3-1 CAN1 arg8::HIS3 HO-Su9-mNeonGreen-KanMX6 ∆cox4::HphNT1 | Intronless mtDNA | This study |
| yFT376 | Su9-mNeonGreen ∆cox4 | Mat a | Mata ade2-1 his3-11,15 trp1-1 leu2-3,112 ura3-1 CAN1 arg8::HIS3 HO-Su9-mNeonGreen-KanMX6 ∆cox4::HphNT1 |  | This study |
| yFT377 | Pgk1-Su9-QUEEN-2m ∆cox4 | Mat alpha | leu2-3,112 trp1-1 can1-100 ura3-1 ade2-1 his3-11,15 LEU-Pgk1-Su9-QUEEN-2M ∆cox4::HphNT1 |  | This study |
| yFT378 | Pgk1-Su9-QUEEN-2m ∆cob::ARG8 ∆cox4 | Mat alpha | ade2-1 his3-11,15 trp1-1 leu2-3,112 ura3-1 CAN1 arg8::HIS3 LEU-Pgk1-Su9-QUEEN-2M ∆cox4::HphNT1 | ∆*cob*::ARG8 intronless mtDNA | This study |
| yFT379 | Pgk1-Su9-QUEEN-2m ∆*cox2*::ARG8 ∆*cox4* | Mat a | ade2-1 his3-11 15 trp1-1 leu2-3 112 ura3-1 CAN1 arg8::HIS3 LEU-Pgk1-Su9-QUEEN-2M ∆cox4::HphNT1 | ∆*cox2*::ARG8M | This study |
| yFT380 | Pgk1-Su9-QUEEN-2m ∆cox4 | Mat a | ade2-1 his3-11,15 trp1-1 leu2-3,112 ura3-1 CAN1 arg8::HIS3 LEU-Pgk1-Su9-QUEEN-2M::URA3 ∆cox4::HphNT1 | Intronless mtDNA | This study |
| yFT381 | Pgk1-Su9-QUEEN-2m ∆atp6::ARG8 ∆cox4 | Mat a | ade2-1 his3-11,15 trp1-1 leu2-3,112 ura3-1 CAN1 arg8::HIS3 LEU-Pgk1-Su9-QUEEN-2M ∆cox4::HphNT1 | ∆atp6::ARG8 | This study |
| yFT382 | Pgk1-Su9-QUEEN-2m ∆cox4 | Mat alpha | Mata ade2-1 his3-11,15 trp1-1 leu2-3,112 ura3-1 CAN1 arg8::HIS3 LEU-Pgk1-Su9-QUEEN-2M ∆cox4::HphNT1 |  | This study |
| yFT383 | Pgk1-Su9-QUEEN-2m ∆cox4 | Mat a | leu2-3,112 trp1-1 can1-100 ura3-1 ade2-1 his3-11,15 LEU-Pgk1-Su9-QUEEN-2M ∆cox4::HphNT1 |  | This study |
| yFT384 | Pgk1-Su9-QUEEN-2m ∆cox4 | Mat alpha | ade2-1 his3-11,15 trp1-1 leu2-3,112 ura3-1 CAN1 arg8::HIS3 LEU-Pgk1-Su9-QUEEN-2M ∆cox4::HphNT1 | Intronless mtDNA | This study |
| yFT385 | Pgk1-Su9-QUEEN-2m ∆cox4 | Mat a | Mata ade2-1 his3-11,15 trp1-1 leu2-3,112 ura3-1 CAN1 arg8::HIS3 LEU-Pgk1-Su9-QUEEN-2M ∆cox4::HphNT1 |  | This study |
| yFT398 | Su9-mNeonGreen ∆rip1 | Mat alpha | leu2-3,112 trp1-1 can1-100 ura3-1 ade2-1 his3-11,15 HO-Su9-mNeonGreen-KanMX6 ∆rip1::HphNT1 |  | This study |
| yFT399 | Su9-mNeonGreen ∆cob::ARG8 ∆rip1 | Mat alpha | ade2-1 his3-11,15 trp1-1 leu2-3,112 ura3-1 CAN1 arg8::HIS3 HO-Su9-mNeonGreen-KanMX6 ∆rip1::HphNT1 | ∆cob::ARG8 | This study |
| yFT400 | Su9-mNeonGreen ∆cox2::ARG8 ∆rip1 | Mat a | ade2-1 his3-11 15 trp1-1 leu2-3 112 ura3-1 CAN1 arg8::HIS3 HO-Su9-mNeonGreen-KanMX6 ∆rip1::HphNT1 | ∆cox2::ARG8 | This study |
| yFT401 | Su9-mNeonGreen ∆rip1 | Mat a | ade2-1 his3-11,15 trp1-1 leu2-3,112 ura3-1 CAN1 arg8::HIS3 HO-Su9-mNeonGreen-KanMX6::URA3 ∆rip1::HphNT1 | Intronless mtDNA | This study |
| yFT402 | Su9-mNeonGreen ∆atp6::ARG8 ∆rip1 | Mat a | ade2-1 his3-11,15 trp1-1 leu2-3,112 ura3-1 CAN1 arg8::HIS3 HO-Su9-mNeonGreen-KanMX6 ∆rip1::HphNT1 | ∆atp6::ARG8 | This study |
| yFT403 | Su9-mNeonGreen ∆rip1 | Mat alpha | Mata ade2-1 his3-11,15 trp1-1 leu2- Mat a 3,112 ura3-1 CAN1 arg8::HIS3 HO-Su9-mNeonGreen-KanMX6 ∆rip1::HphNT1 |  | This study |
| yFT404 | Su9-mNeonGreen ∆rip1 | Mat a | leu2-3,112 trp1-1 can1-100 ura3-1 ade2-1 his3-11,15 HO-Su9-mNeonGreen-KanMX6 ∆rip1::HphNT1 |  | This study |
| yFT405 | Su9-mNeonGreen ∆rip1 | Mat alpha | ade2-1 his3-11,15 trp1-1 leu2-3,112 ura3-1 CAN1 arg8::HIS3 HO-Su9-mNeonGreen-KanMX6 ∆rip1::HphNT1 |  | This study |
| yFT406 | Su9-mNeonGreen ∆rip1 | Mat a | Mata ade2-1 his3-11,15 trp1-1 leu2-3,112 ura3-1 CAN1 arg8::HIS3 HO-Su9-mNeonGreen-KanMX6 ∆rip1::HphNT1 |  | This study |
| yFT407 | Pgk1-Su9-QUEEN-2m ∆rip1 | Mat alpha | leu2-3,112 trp1-1 can1-100 ura3-1 ade2-1 his3-11,15 LEU-Pgk1-Su9-QUEEN-2M ∆rip1::HphNT1 |  | This study |
| yFT408 | Pgk1-Su9-QUEEN-2m ∆cob::ARG8 ∆rip1 | Mat alpha | ade2-1 his3-11,15 trp1-1 leu2-3,112 ura3-1 CAN1 arg8::HIS3 LEU-Pgk1-Su9-QUEEN-2M ∆rip1::HphNT1 | ∆cob::ARG8 | This study |
| yFT409 | Pgk1-Su9-QUEEN-2m ∆cox2::ARG8 ∆rip1 | Mat a | ade2-1 his3-11 15 trp1-1 leu2-3 112 ura3-1 CAN1 arg8::HIS3 LEU-Pgk1-Su9-QUEEN-2M ∆rip1::HphNT1 | ∆cox2::ARG8 | This study |
| yFT410 | Pgk1-Su9-QUEEN-2m ∆rip1 | Mat a | ade2-1 his3-11,15 trp1-1 leu2-3,112 ura3-1 CAN1 arg8::HIS3 LEU-Pgk1-Su9-QUEEN-2M::URA3 ∆rip1::HphNT1 | Intronless mtDNA | This study |
| yFT411 | Pgk1-Su9-QUEEN-2m ∆atp6::ARG8 ∆rip1 | Mat a | ade2-1 his3-11,15 trp1-1 leu2-3,112 ura3-1 CAN1 arg8::HIS3 LEU-Pgk1-Su9-QUEEN-2M ∆rip1::HphNT1 | ∆atp6::ARG8 | This study |
| yFT412 | Pgk1-Su9-QUEEN-2m ∆rip1 | Mat alpha | Mata ade2-1 his3-11,15 trp1-1 leu2-3,112 ura3-1 CAN1 arg8::HIS3 LEU-Pgk1-Su9-QUEEN-2M ∆rip1::HphNT1 |  | This study |
| yFT413 | Pgk1-Su9-QUEEN-2m ∆rip1 | Mat a | leu2-3,112 trp1-1 can1-100 ura3-1 ade2-1 his3-11,15 LEU-Pgk1-Su9-QUEEN-2M ∆rip1::HphNT1 |  | This study |
| yFT414 | Pgk1-Su9-QUEEN-2m ∆rip1 | Mat alpha | ade2-1 his3-11,15 trp1-1 leu2-3,112 ura3-1 CAN1 arg8::HIS3 LEU-Pgk1-Su9-QUEEN-2M ∆rip1::HphNT1 | Intronless mtDNA | This study |
| yFT415 | Pgk1-Su9-QUEEN-2m ∆rip1 | Mat a | Mata ade2-1 his3-11,15 trp1-1 leu2-3,112 ura3-1 CAN1 arg8::HIS3 LEU-Pgk1-Su9-QUEEN-2M ∆rip1::HphNT1 |  | This study |
| \| yFT515 \| \| --- \| \|  \| \|  \| | Pgk1-QUEENII | Mat a (haploid) | ade2-1 his3-11,15 trp1-1 leu2-3,112 ura3-1 CAN1 arg8::HIS3 cob::ARG8M Pgk1-QUEENII | ∆cob::ARG8 intronless | This study |
| yFT516 | Pgk1-QUEENII | Mat alpha (haploid) | ade2-1 his3-11 15 trp1-1 leu2-3 112 ura3-1 CAN1 arg8::HIS3 cox2::ARG8M Pgk1-QUEENII | ∆cox2::ARG8M | This study |
| \| yFT517 \| \| --- \| \|  \| \|  \| | Pgk1-QUEENII | Mat a (haploid) | ade2-1 his3-11,15 trp1-1 leu2-3,112 ura3-1 CAN1 arg8::HIS3 Pgk1-QUEENII | Intronless mtDNA | This study |
| yFT518 | Pgk1-QUEENII | Mat a (haploid) | Mata ade2-1 his3-11,15 trp1-1 leu2-3,112 ura3-1 CAN1 arg8::HIS3 Pgk1-QUEENII |  | This study |
| \| yFT519 \| \| --- \| \|  \| \|  \| | Pgk1-QUEENII | Mat a (haploid) | ade2-1 his3-11,15 trp1-1 leu2-3,112 ura3-1 CAN1 arg8::HIS3 ∆atp6::ARG8 Pgk1-QUEENII | ∆atp6::ARG8 | This study |
| yFT520 | rho0 Su9-mNeonGreen::G418 | Mat a (haploid) | Mata ade2-1 his3-11,15 trp1-1 leu2-3,112 ura3-1 CAN1 arg8::HIS3 HO-Su9-mNeonGreen::G418 | rho0 | This study |
| yFT521 | Pgk1-Su9-QUEENII Su9-mKate2::Ura3 | Mat a (haploid) | Mata ade2-1 his3-11,15 trp1-1 leu2-3,112 ura3-1 CAN1 arg8::HIS3 LEU-Pgk1-Su9-QUEENII HO-Su9-mKate2 |  | This study |
