# Supplementary material for: Local mitochondrial physiology defined by mtDNA quality guides purifying selection
Source: PLoS Genet. 2026 Jan 9;22(1):e1011836. doi: 10.1371/journal.pgen.1011836 (PMC12810922; doi:10.1371/journal.pgen.1011836)
Supplement: S2 Table — (DOCX) [file pgen.1011836.s002.docx]

Table 2 - Plasmids used in this study

| Name | Insert | Selection marker | Origin |
| --- | --- | --- | --- |
| pCO074 | pfa6a-hphNT1 | Hygromycin | Janke et al. (2004) |
| pCO442 | HO-Su9-mNeonGreen | G418 | Jakubke et al. (2021) |
| pFT025 | LEU-mtQueen-2m | URA3 | This study |
| pFT031 | LEU-cytQueen-2m | URA3 | This study |
