# Supplementary figures and images for: Local mitochondrial physiology defined by mtDNA quality guides purifying selection
Source: PLoS Genet. 2026 Jan 9;22(1):e1011836. doi: 10.1371/journal.pgen.1011836 (PMC12810922; doi:10.1371/journal.pgen.1011836)

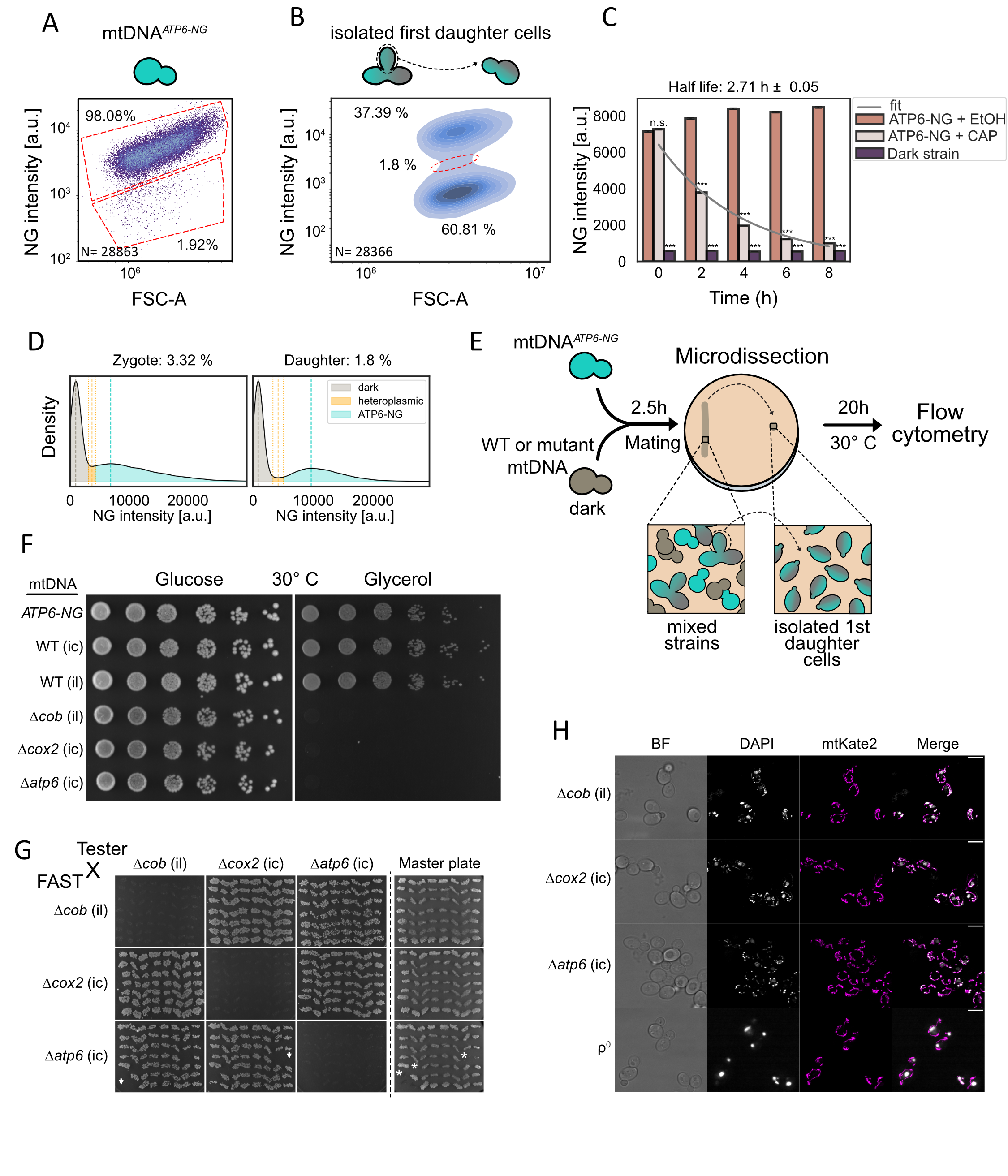

Supplement: S1 Fig — (A) Scatterplot of the data shown in Fig 1B, illustrating the minor fraction of dark cells. The population of green fluorescent cells is highlighted by the red circle. (B) KDE plot of FAST analysis of mtDNAATP6-NG mated with mtDNAic when the first daughter cell of the Zygote was picked. The heteroplasmic cell population is highlighted by the red circle. (C) Histogram of fluorescence intensity levels of mtDNAATP6-NG cells with a PDR5 deletion following 1mg/mL chloramphenicol (CAP) treatment. Atp6-NG fluorescence half-life was determined by fitting an exponential decay to the log-transformed fluorescence medians over time. (D) Histogram of the NG intensity distribution among FAST population after 20 h of mtDNAATP6-NG mated with mtDNAic when zygotes or daughters of the zygotes were picked. Dotted lines mark the two peak maxima and the interpeak valley; shaded regions denote population assignments. The heteroplasmic window is defined symmetrically around the valley with a width of ±10 % of the interpeak distance. (E) Schematic of the modified FAST workflow: instead of harvesting zygotes, first-division daughters are detached and relocated on agar. (F) Drop-dilution assay of mtDNA-variant strains on fermentable (glucose) and non-fermentable (glycerol) rich media. Plates were incubated at 30 °C and imaged after 48 h. (G) Crosses of mtDNAΔcob, mtDNAΔcox2, and mtDNAΔatp6 strains against tester strains to screen for secondary deletions in the mitochondrial genome. Crosses were replicaplated on non-fermentable medium (YPG) to assess growth rescue. The right panels show the corresponding master plate of streaked cells on SC-Arg. White asterisks indicate colonies that failed to grow and were excluded from analysis; white arrows indicate crosses that did not rescue growth on YPG. (H) Representative images of DAPI stainings of mtDNAΔcob, mtDNAΔcox2, mtDNAΔatp6, and ρ0 cells expressing mtKate2 as a mitochondrial marker, used to visualize mtDNA. Note that no ρ0 cells were identifie [file pgen.1011836.s003.tiff]

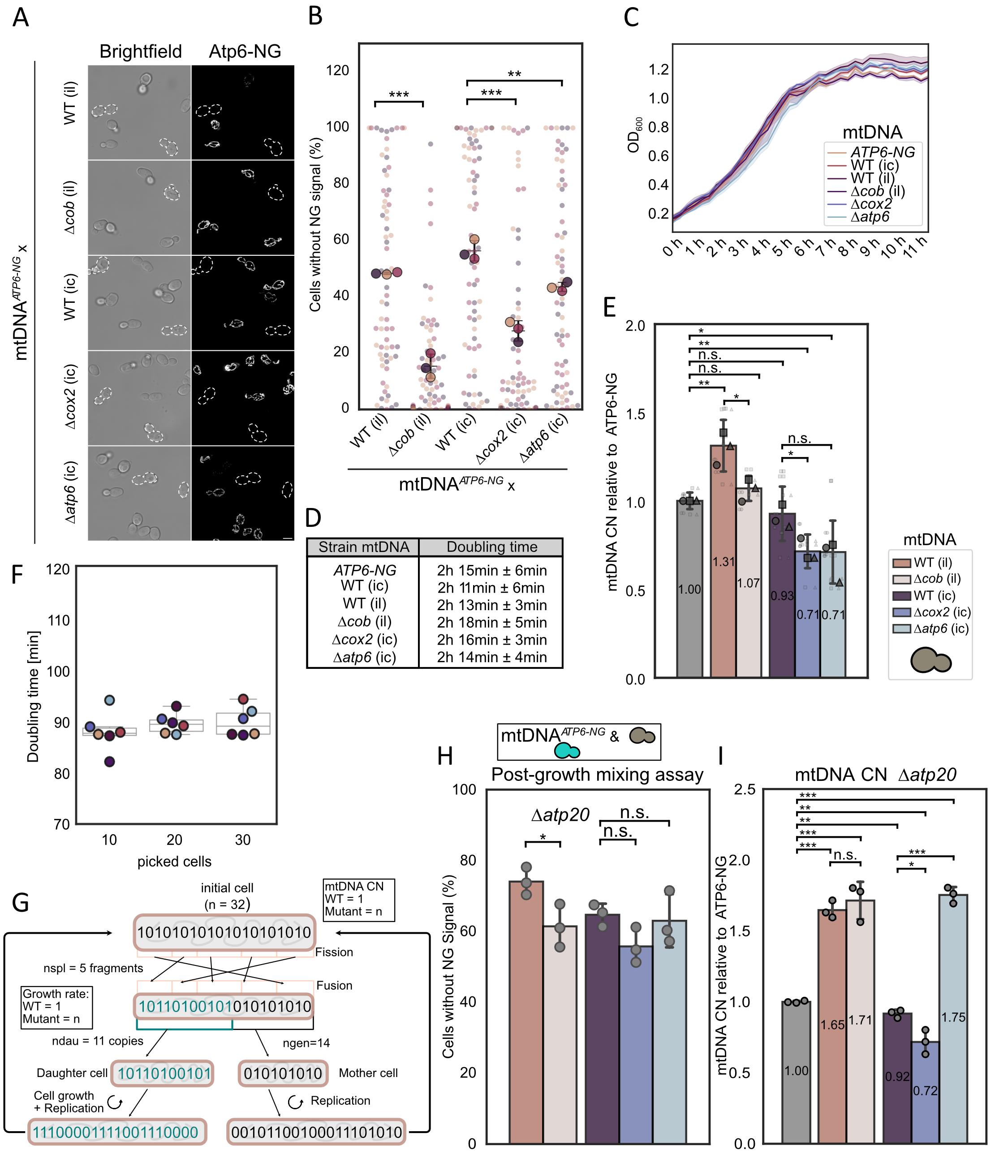

Supplement: S2 Fig — (A) Representative widefield fluorescence microscopy images of microcolonies grown for 20 h following isolation of 30 first-division daughters from mtDNAATP6-NG×mtDNAic, mtDNAΔcob, mtDNAil, mtDNAΔcox2, or mtDNAΔatp6 matings. (B) Microscopy-based FAST quantification: individual daughter cells were arrayed on distinct plate positions, enabling each to form a microcolony over 20 h; colonies were then relocated to separate wells for imaging. Approximately 200 cells per well were scored for fluorescence status, with the ratio of fluorescent to non-fluorescent cells plotted as individual points in a swarmplot. The three big dots represent the mean of a single replicate. (C) Growth curves of all FAST strains: the solid curve represents the mean trajectory of three independent biological replicates, and the shaded region indicates the 95% confidence interval. (D) Mean doubling times for each strain, calculated from the growth curves in (C); errors represent one standard deviation (SD) across three biological replicates. (E) mtDNA copy number (CN) of FAST strains relative to the mtDNAATP6-NG strain, determined via qPCR. qPCR experiments were performed three times, each with three biological replicates and three technical replicates per biological replicate. (F) Doubling time of mtDNAATP6-NG cells grown on solid medium. Either 10, 20, or 30 individual cells were relocated to defined positions on the agar plate and cultured for 20 hours to allow microcolony formation. Subsequently, the resulting colonies were independently harvested into 100 μL of SC medium, and cell numbers were quantified by flow cytometry. Doubling times were calculated based on the known initial and final cell counts. Each data point represents a biological replicate. (G) Schematic of the workflow of the simulation used for Figs 2G and 4F. (H) Post-growth mixing assay of haploid Δatp20 cells. (I) mtDNA CN in Δatp20 cells relative to the mtDNAATP6-NG strain. Panels (B),(E),(H) and (I) were analysed for stat [file pgen.1011836.s004.tiff]

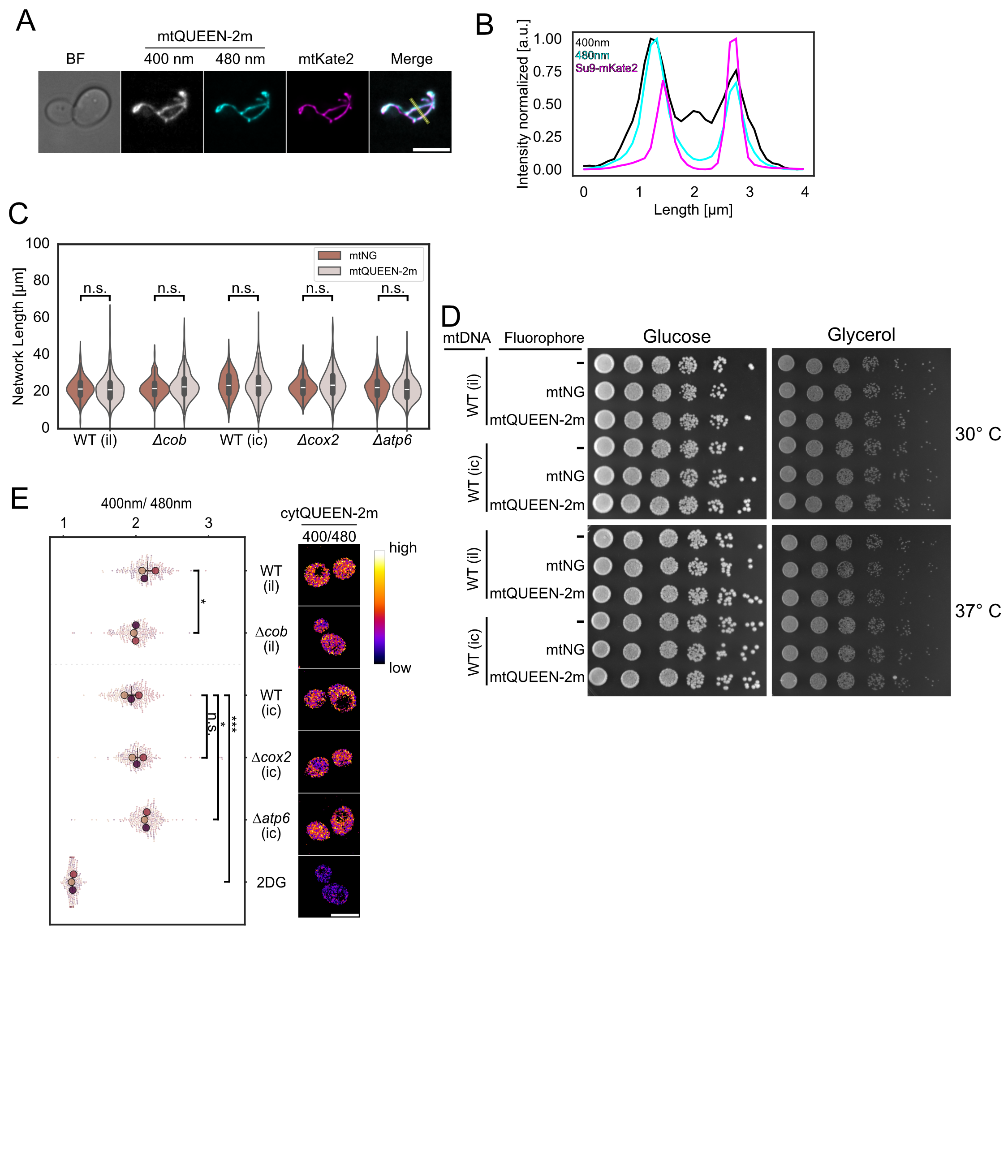

Supplement: S3 Fig — (A) Maximum-intensity projections of widefield fluorescence microscopy images of a cell expressing mtQUEEN-2m and mtKate2, with corresponding (B) line profiles of the respective fluorescent channels. The yellow line with a white arrow in the “Merge” panel indicates the pixel positions used for intensity profiling. (C) Comparison of mitochondrial network length in cells expressing mtNG or mtQUEEN-2m. (D) Drop-dilution assay of mtDNAil and mtDNAic cells expressing no fluorophore, mtNG, or mtQUEEN-2m. Cells were spotted onto YPD (glucose) or YPG (glycerol) plates and incubated for 48 h at either 30°C or 37°C. (E) Quantification of relative cytosolic ATP levels in FAST strains expressing cytQUEEN-2m, shown together with a representative ratiometric image. Scale bars in (A) and (E) represent 5 μm. Panels (C) and (E) were analysed for statistical significance using an unpaired Student’s t-test performed on replicate means (*P < 0.05; **P < 0.005; ***P < 0.001). (TIFF) [file pgen.1011836.s005.tiff]

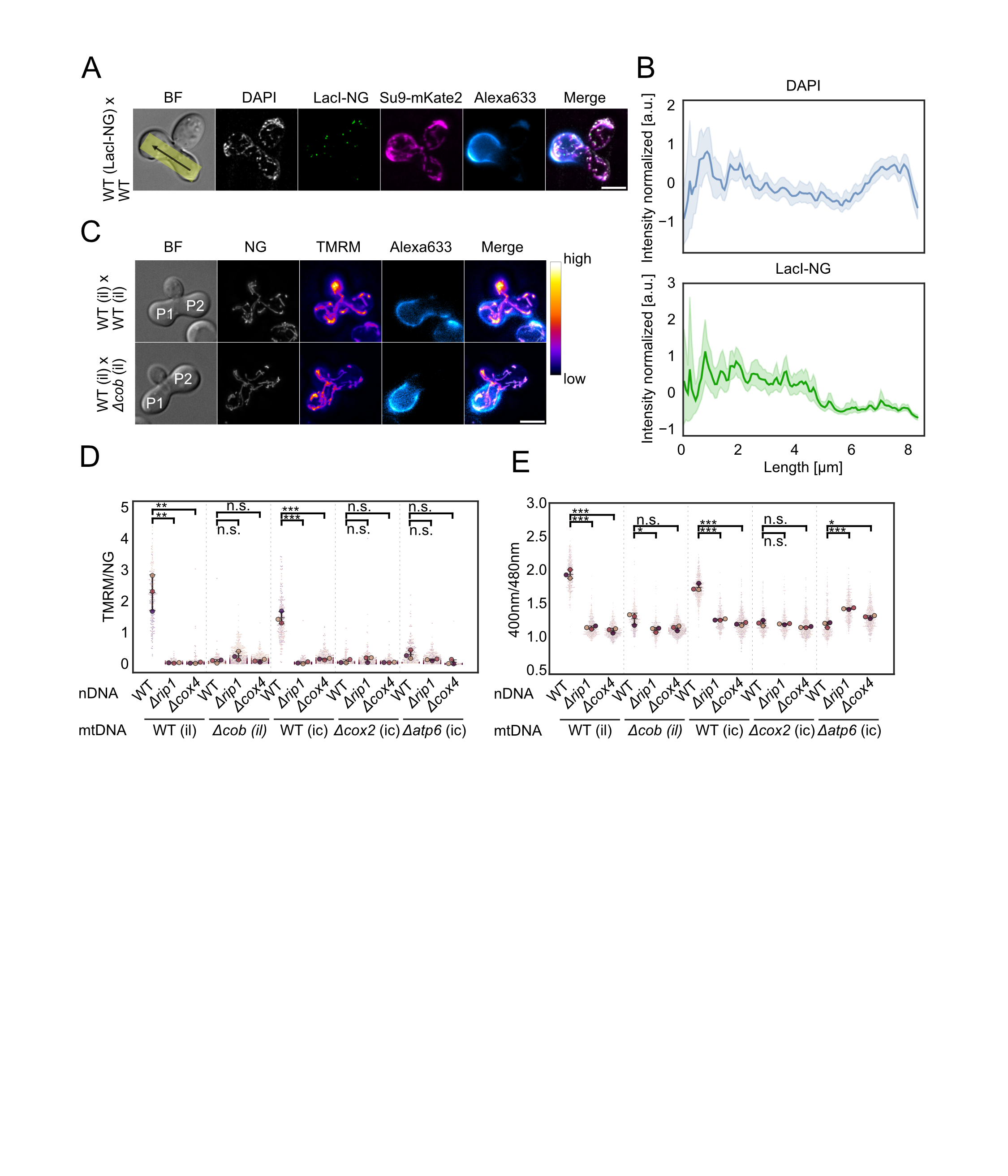

Supplement: S4 Fig — (A) Maximum-intensity projection of a widefield fluorescence microscopy image of a Zygote. One parental cell expresses LacI-3xmNeongreen that can bind to the LacO repeats integrated in its mtDNA and mitochondrial targeted mKate2. Prior to mating its cell wall was stained with conA-Alexa633. The other cell does not express any fluorophores and its mtDNA lacks LacO repeats. Zygotes were stained with 2μg/ml DAPI to visualize the entire mtDNA pool of the cell. Note that LacI-3xmNeongreen spots are only observed in one parental cell. (B) Line profiles of DAPI and Laci-NG intensities of 50 Zygotes with a line width of 41 pixels as depicted in the BF panel of panel (A). Intensities were each normalized by z-score scaling (C) Representative widefield fluorescence micrographs of zygotes expressing mtNG (corresponding to Fig 3G). Zygotes were stained with 50nM TMRM to assess ΔΨ. Parental cells contain either the same mitochondrial genome (upper row) or comprise WT and mutant mtDNA; P1 and P2 cells are indicated in the bright-field panel. Prior to mating, P1 cell walls were labeled with conA-Alexa633. Superplots of (D) membrane potential levels or (E) mitochondrial ATP levels in zygotes of WT, Δrip1, and Δcox4 nuclear backgrounds. The three big dots represent the mean of each replicate and small dots represent individual-cell values. Scale bar in (A) and (C) = 5μM. Panels (D) and (E) were analysed for statistical significance using an unpaired student T-test was performed on the means of the replicates (*P <0.05; **P <0.005; ***P < 0.001). (TIFF) [file pgen.1011836.s006.tiff]

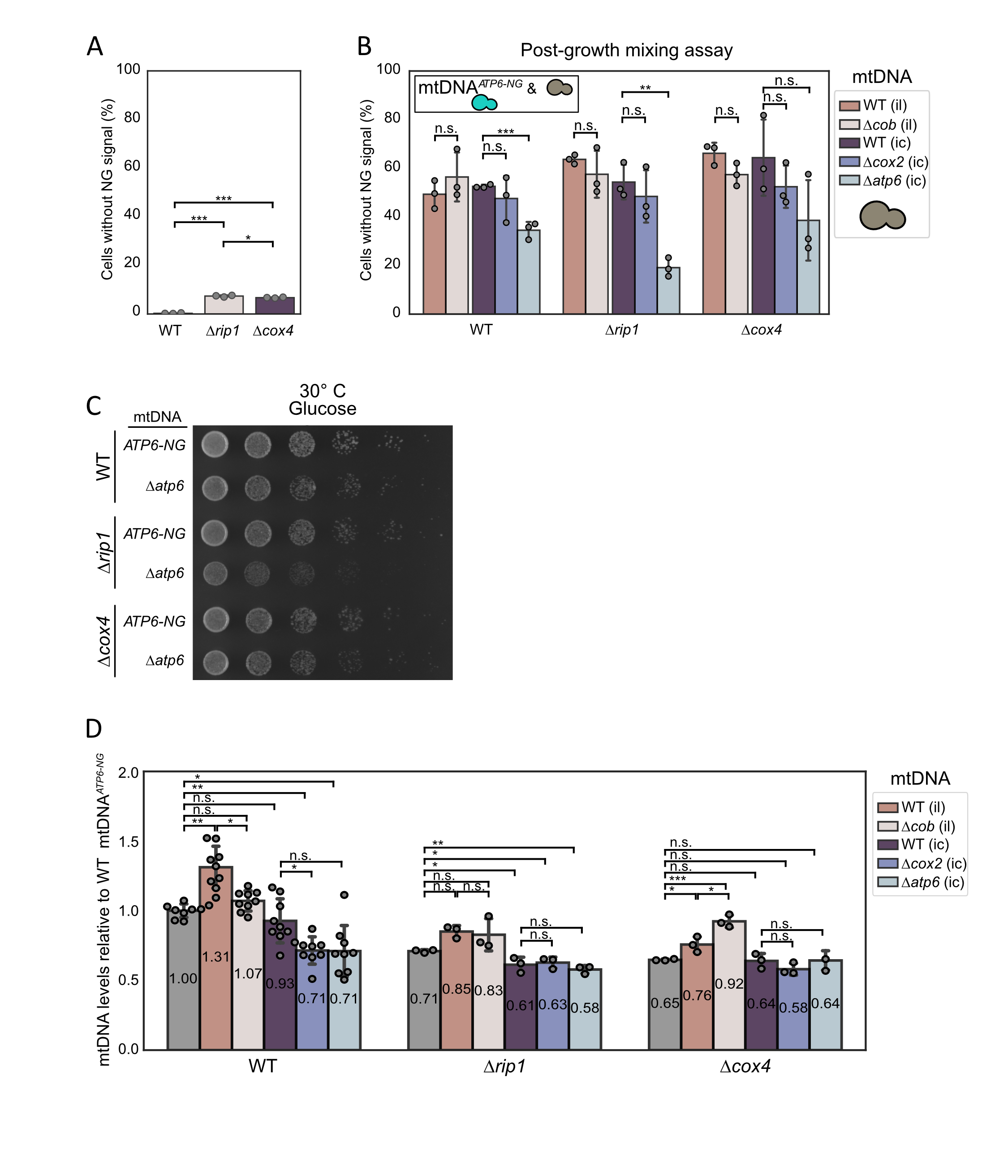

Supplement: S5 Fig — (A) Fraction of cells within a mtDNAATP6-NG population without NG signal (B) Post-growth mixing assay of Δrip1 and Δcox4 nuclear-background strains compared to WT. (C) Drop dilution assay of mtDNAATP6-NG and mtDNAΔatp6 strains in the WT, Δrip1 and Δcox4 nuclear background. Serial dilutions were spotted on YPD and incubated for 24 h at 30 °C. (D) mitochondrial copy numbers relative to WT mtDNAATP6-NG for Δrip1 and Δcox4 strains. Panels (A), (B) and (C) were analysed for statistical significance using an unpaired student T-test was performed on the means of the replicates (*P <0.05; **P <0.005; ***P < 0.001). (TIFF) [file pgen.1011836.s007.tiff]
