# Supplementary material for: Local mitochondrial physiology defined by mtDNA quality guides purifying selection
Source: PLoS Genet. 2026 Jan 9;22(1):e1011836. doi: 10.1371/journal.pgen.1011836 (PMC12810922; doi:10.1371/journal.pgen.1011836)
Supplement: Attachment — Submitted filename: Response to Reviewers.pdf [file pgen.1011836.s009.pdf]

LMU
LUDWIG-
MAXIMILIANS-
UNIVERSITÄT
MÜNCHEN

FACULTY OF BIOLOGY
FAKULTÄT FÜR BIOLOGIE

Prof. Dr. Christof Osman · Großhaderner Str. 2 · 82131 Planegg-Martinsried

Prof. Dr. Christof Osman

Ludwig-Maximilians-Universität
Biocenter of the LMU
Großhaderner Str. 2
82152 Planegg-Martinsried

☎ (+49) 89-2180-74756
✉ osman@bio.lmu.de

December 8, 2025

Response to reviewer comments regarding our manuscript entitled: 'Local Mitochondrial Physiology Defined by mtDNA Quality Guides Purifying Selection'

Dear Miguel,

We are delighted to receive positive feedback on our manuscript titled "Local Mitochondrial Physiology Defined by mtDNA Quality Guides Purifying Selection". We appreciate the time and effort you and the reviewers have dedicated to evaluating our submission. We are grateful for the comments and we believe that our additional work inspired by the comments has improved the manuscript. We have highlighted all changes in the revised version of the manuscript. In the following, you will find a point-by-point response to the reviewers comments.

Reviewer #1:

While I think that additional experiments are not necessary at this stage, it would be great if the authors could in greater depth discuss possible molecular mechanisms of selection. They discuss that mitochondrial transport into the bud could be one possible way to exclude mutant mtDNA from inheritance. However, as suggested in a recent study (PMID: 40911417), it is also possible that the improved bioenergetic state around intact mtDNA could support replication of this DNA, while bioenergetic problems surrounding mutant mtDNA could lead to lower efficiency of replication and hence a competitive disadvantage.

We thank Reviewer #1 for the very positive evaluation and for highlighting this important aspect. Indeed, the molecular mechanisms by which mutant mtDNA is selectively purged from populations remain a fascinating and unresolved question. In the revised manuscript, we have included the suggested

reference (PMID: 40911417) in the section where we discuss potential mechanisms of selection.

Reviewer #2:

— What is the half-life of ATP6-NG? This question is crucial for the entire study because if ATP6-NG has a sufficiently long half-life, the intensity of the NG signal can be explained by inheritance of the protein from mother cells, retained even several generations after the mtDNA ATP6-NG was displaced by mtDNA without the fluorescent protein. It should be noted that mitochondrial inner membrane proteins usually have extremely long half-lives. I suggest assessing the ATP6-NG half-life by incubating cells in the presence of a mitochondrial translation inhibitor (e.g., erythromycin) and analyzing the distribution of signal levels across the population at different time points.

We thank the reviewer for raising this important point. Indeed, a high stability of Atp6-NG could in principle lead to an overestimation of the fraction of cells containing $mtDNA^{Atp6\text{-}NG}$, if the fluorescent signal persisted after the corresponding mtDNA variant had been lost.

In the revised manuscript, we have now determined the half-life of the fluorescent signal of Atp6-NG by incubating cells with chloramphenicol to inhibit mitochondrial translation and monitoring the decay of the existing Atp6-NG signal over time (S1 FigC of the revised manuscript). Consistent with our previous findings, we estimate a fluorescent half-life of approximately 2.7 hours. Comparison of Atp6-NG fluorescence in $mtDNA^{Atp6\text{-}NG}$ cells after 2 h, 4 h, 6 h and 8 h of chloramphenicol treatment with untreated controls revealed a progressive signal decrease, such that after 4 h the fluorescence intensity was clearly reduced and distinguishable from untreated cells.

These data indicate that only cells that have lost $mtDNA^{Atp6\text{-}NG}$ within the last cell cycle would still appear Atp6-NG–positive in our assays. Moreover, as shown previously (Roussou et al., 2024), populations derived from heteroplasmic founder cells almost completely segregate within 20 h. Together, these results demonstrate that any residual fluorescence due to stable Atp6-NG protein is likely to have only a minor impact on our quantifications. We have included the results and a discussion of these new experiments in the revised manuscript.

— As an addition to the previous question, I would like to point to Figure 1B, which shows the absence of even a small subpopulation of NG-negative cells, while W303 strain used in this study usually contains 2–5% petite cells in suspension, which are likely unable to perform mitochondrial protein synthesis. Why is there no such population?

We thank the reviewer for this observation. Indeed, NG-negative cells are present in our samples, but they are not readily visible in the kernel density estimate (KDE) representation of Figure 1B because their frequency is very low and the density smoothing underrepresents rare events. To make this clearer, we have now added a supplementary figure showing the raw flow-cytometry data at the single-cell level (S1 FigA of the revised manuscript). In this representation, we highlight a small subpopulation—approximately 1.9 %—that lacks detectable Atp6-NG signal.

This percentage is lower than the typical fraction of petite cells observed in growing W303 cultures (about 5 %). This likely is explained by the fact that petite cells can still synthesize Atp6-NG if their mtDNA carries deletions or mutations in genes other than *ATP6*. We have clarified this point in the revised Results section and refer to the new supplementary figure for visualization.

— How stable is mtDNA with deletions of ATP6, COB, and COX2? Secondary deletions can emerge in yeast rho– cells; could this be the case for these strains? It is also theoretically possible that rho– cells lose their mtDNA completely and become rho0. This process is favored because dysfunctional mtDNA no longer provides a fitness advantage to the host cell, and the complete loss of mtDNA can even free up resources required for mtDNA replication. I suggest checking the percentage of rho0 cells (e.g., those with DAPI-negative cytoplasm) in strains harboring deletions in mtDNA. Otherwise, the increase in NG-positive cells observed in mtDNA-ATP6NG X mtDNA deltaCOB crossings could be explained by a large fraction of rho0 cells in mtDNA deltaCOB (and in other deletion strains in a similar way). Although, Figure 4E shows that deletion of nuclear-encoded OXPHOS genes removes the difference between mtDNA-ATP6NG X mtDNA deltaCOB and mtDNA-ATP6NG X mtDNA WT crossings, it would be better to verify this with a direct control.

We thank the reviewer for this valuable suggestion. We have now performed DAPI staining of strains carrying mtDNA deletions in *COB*, *COX2*, or *ATP6*. In these stainings, we did not observe any ρ^0 cells lacking mtDNA entirely. The images are included in Figure S1H of the revised manuscript.

To further evaluate the stability of these mtDNA variants, we examined the presence of ρ^- cells by crossing 56 colonies derived from single cells of mtDNA$^{\Delta cob}$, mtDNA$^{\Delta cox2}$, or mtDNA$^{\Delta atp6}$ strains with cells containing the respective other two mtDNA variants. In all mtDNA$^{\Delta cob}$ and mtDNA$^{\Delta cox2}$ cells, 100% of the resulting diploid offspring were able to grow on non-fermentable carbon sources, indicating that respiration was restored by complementation between the mtDNA variants. In mtDNA$^{\Delta atp6}$ cells, only two colonies failed to complement the other deletions. This analysis demonstrates that virtually all single-colony parental strains retained mtDNA capable of complementation. This analysis is now presented in Figure S1G of the revised manuscript.

While we cannot exclude secondary deletions outside the targeted genes, these results collectively indicate that the mtDNA$^{\Delta cob}$, mtDNA$^{\Delta cox2}$, and mtDNA$^{\Delta atp6}$ variants are stably maintained, with low frequencies of ρ^- or ρ^0 cells.

— Does deletion of COX2, ATP6, and COB affect the copy number of mtDNA? If these deletions reduce mtDNA levels in haploid cells, this could explain the main result without invoking the quality control mechanism hypothesis. The mtDNA copy number should be assessed because a pronounced difference between the studied strains would challenge the main conclusion of the study.

We thank the reviewer for highlighting this point. It was an excellent and crucial suggestion that had not been addressed sufficiently in our original submission. In response, we quantified mtDNA copy numbers in haploid strains carrying either intact mtDNA or mtDNA variants lacking *COX2*, *ATP6*, or *COB*. This analysis showed that mtDNA molecules harboring deletions of protein-coding genes are indeed present at modestly reduced copy numbers compared to their intact counterparts in haploid cells containing exclusively one mtDNA variant (revised Fig. S2E).

To evaluate whether such differences could account for the FAST results, we incorporated the experimentally measured mtDNA copy numbers together with strain-specific growth rates into our simulations. These analyses revealed that copy-number differences and growth defects can cause shifts in the expected distribution of progeny inheriting mutant versus intact mtDNA. Critically, they do not reproduce the strong depletion of mtDNA$^{\Delta cob}$ or, to a minor extent, mtDNA$^{\Delta cox2}$ observed experimentally (revised

Fig. 2F,G), supporting additional intracellular selection against these variants. In contrast, the behaviour of mtDNA$^{\Delta atp6}$ is well explained by these parameters alone and the simulations recaptiulate the findings of the FAST analysis, indicating minimal intracellular selection against mtDNA$^{\Delta atp6}$.

We also quantified mtDNA copy numbers in strains lacking *ATP20* (revised fig S2I), *RIP1*, or *COX4* (revised Fig S5 D), which are used in additional FAST experiments. For $\Delta rip1$ and $\Delta cox4$ backgrounds, simulations that incorporate both mtDNA copy number and growth rates now align closely with the experimental FAST data, supporting the conclusion that intracellular selection against mtDNA$^{\Delta cob}$ and mtDNA$^{\Delta cox2}$ is abolished in these mutants (Fig 4F in the revised manuscript).

These new data significantly strengthen the study. They allow us to attribute purifying selection to distinct modes: for mtDNA$^{\Delta atp6}$, selection is largely explained by mtDNA abundance and growth defects, whereas for mtDNA$^{\Delta cob}$ and mtDNA$^{\Delta cox2}$, an additional mechanism—independent of these factors—must operate, consistent with intracellular selection. We have revised the abstract, introduction, and discussion to reflect these insights.

Finally, based on the new copy-number data, we removed the asymmetric mating experiments involving only one parent lacking *COX4* or *RIP1*. These datasets added interpretive complexity that could not be resolved with respect to mtDNA copy number and in our view do not strengthen the manuscript.

We hope that the additional experiments and analyses fully satisfy the reviewer's important concern regarding mtDNA copy number.

— To verify that the observed effects are not due to differences in cell-level fitness of the mutant variants, the authors performed a "post-growth mixing assay" (Figure 2C, E). However, the conditions in the mixed and clonal cultures of wild-type and OXPHOS-deficient cells might differ significantly. I wonder why the authors did not mix mtDNAATP6-NG cells and "Dark" cells prior to their growth on the plate (using same mating type cells). This approach would more closely mimic the conditions of the heteroplasmic zygote experiment.

We thank the reviewer for this suggestion. It is of course possible that the growth behavior of haploid cells differs when they are part of a mixed population. To test this, we isolated 30 haploid cells each from mtDNA$^{ATP6\text{-}NG}$ and either mtDNA^{il} or mtDNA$^{\Delta cob}$ of the same mating type, placed them at the same position on a YPD plate, and—just as in the post-growth mixing assay—measured fluorescence by flow cytometry after 20 hours. We did not observe major differences compared to our post-growth mixing assay (Figure 1), and therefore decided to retain the latter in the manuscript. However, we would be happy to include it in the manuscript if requested by the reviewer or editor.

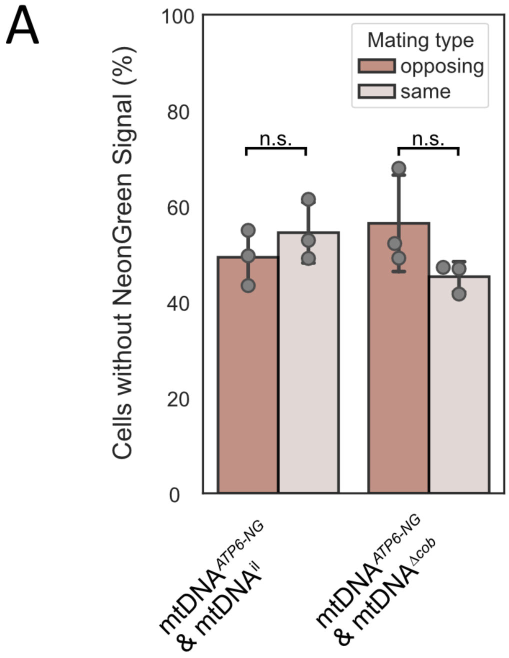

Figure 1: **Figure for the reviewers.** Comparison of post growth mixing assay with of cells with same vs opposing mating type.

— Figure S4-A the fact that delta rip1 and delta cox4 shows statistical significant difference in NG signal is confusing. Can it be a mistake?

We thank the reviewer for this comment. We carefully re-checked the raw data, analysis code, and statistical tests, and confirmed that the observed difference between Δ*rip1* and Δ*cox4* is correct and not the result of an error. Although the numerical difference between these strains may appear small, both groups display very low internal variability, and their measurements do not overlap. This results in a statistically significant difference despite the small effect size.

Reviewer #3:

However, this reviewer considers unsound the evaluation of OXPHOS mutants based on measurements of cellular ATP levels using the probe QUEEN-2. In most cells, phosphorylation potential (ΔGp) is sustained by both cytosolic and mitochondrial ATP production. In contrast, S. cerevisiae grown in high glucose undergoes the Crabtree effect, where the high glycolytic flux (ethanol fermentation) should maintain ATP levels independently of OXPHOS. Indeed, the authors clearly show that all mtDNA mutants exhibit identical growth rates, which strongly indicates the absence of ATP limitations that could reduce ΔGp. Measurements of cellular ATP levels under fermentative conditions cannot be used to draw conclusions about OXPHOS function.

We thank the reviewer for raising this important point. In our experiments, we used a construct encoding the QUEEN-2m ATP sensor fused to the mitochondrial targeting sequence (denoted Su9), resulting in efficient import of the sensor into mitochondria (mtQUEEN-2m). Thus, the signal we measured reflects *mitochondrial* rather than total cellular ATP levels. We have clarified this distinction in both the Results and Materials and Methods sections of the revised manuscript. Additionally, we examined whether mtQUEEN-2m has any negative effects on cellular or mitochondrial fitness. Neither spot tests on glycerol nor analyses of the mitochondrial network revealed any disadvantages in cells expressing mtQUEEN-2m (S3 FigC and D of the revised manuscript).

To substantiate this point, we now provide an additional figure demonstrating the correct mitochondrial localization of the mtQUEEN-2m construct by co-localization with a mitochondrial marker (S3 FigA and B of the revised manuscript). Furthermore, to address the reviewer's concern directly, we expressed QUEEN-2m lacking the mitochondrial targeting sequence to monitor cytosolic ATP levels (cytQUEEN-2m). As anticipated, and consistent with the reviewer's argument, we observed no drastic differences in cytosolic ATP levels among the mtDNA mutant strains (S3 FigE of the revised manuscript).

We hope that these clarifications and additional data address the reviewer's concern and strengthen the interpretation of our results.

We hope that our revisions and clarifications address the reviewers' concerns satisfactorily and that the revised manuscript will now be suitable for publication in *PLOS Genetics*. We sincerely thank the reviewers and editors for their constructive feedback, which has helped us to significantly improve the manuscript.

Best regards,

Christof and Felix
